# Antioxidant micronutrients in the critically ill: a systematic review and meta-analysis

**DOI:** 10.1186/cc11316

**Published:** 2012-04-25

**Authors:** William Manzanares, Rupinder Dhaliwal, Xuran Jiang, Lauren Murch, Daren K Heyland

**Affiliations:** 1 Intensive Care Unit, Department of Critical Care Medicine, Universidad de la República, Hospital de Clínicas (University Hospital), Faculty of Medicine, Avda Italia s/n 14th Floor, Montevideo, 11600, Uruguay; 2 Clinical Evaluation Research Unit, Kingston General Hospital, Angada 4, Kingston General Hospital, 76 Stuart Street, Kingston ON, K7L 2V7, Canada; 3 Department of Medicine, Queen's University Kingston, Angada 4, Kingston General Hospital, 76 Stuart Street, Kingston, Ontario K7L 2V7, Canada

## Abstract

**Introduction:**

Critical illness is characterized by oxidative stress, which is a major promoter of systemic inflammation and organ failure due to excessive free radical production, depletion of antioxidant defenses, or both. We hypothesized that exogenous supplementation of trace elements and vitamins could restore antioxidant status, improving clinical outcomes.

**Methods:**

We searched computerized databases, reference lists of pertinent articles and personal files from 1980 to 2011. We included randomized controlled trials (RCTs) conducted in critically ill adult patients that evaluated relevant clinical outcomes with antioxidant micronutrients (vitamins and trace elements) supplementation versus placebo.

**Results:**

A total of 21 RCTs met inclusion criteria. When the results of these studies were statistically aggregated (n = 20), combined antioxidants were associated with a significant reduction in mortality (risk ratio (RR) = 0.82, 95% confidence interval (CI) 0.72 to 0.93, *P *= 0.002); a significant reduction in duration of mechanical ventilation (weighed mean difference in days = -0.67, 95% CI -1.22 to -0.13, *P *= 0.02); a trend towards a reduction in infections (RR= 0.88, 95% CI 0.76 to 1.02, *P *= 0.08); and no overall effect on ICU or hospital length of stay (LOS). Furthermore, antioxidants were associated with a significant reduction in overall mortality among patients with higher risk of death (>10% mortality in control group) (RR 0.79, 95% CI 0.68 to 0.92, *P *= 0.003) whereas there was no significant effect observed for trials of patients with a lower mortality in the control group (RR = 1.14, 95% 0.72 to 1.82, *P *= 0.57). Trials using more than 500 μg per day of selenium showed a trend towards a lower mortality (RR = 0.80, 95% CI 0.63 to 1.02, *P *= 0.07) whereas trials using doses lower than 500 μg had no effect on mortality (RR 0.94, 95% CI 0.67 to 1.33, *P *= 0.75).

**Conclusions:**

Supplementation with high dose trace elements and vitamins may improve outcomes of critically ill patients, particularly those at high risk of death.

## Introduction

Critical illness is characterized by hyperinflammation, cellular immune dysfunction, oxidative stress and mitochondrial dysfunction [1]. Oxidative stress is defined as a state in which the level of toxic reactive oxygen intermediates overcome the endogenous antioxidant defenses of the host and damage biologically relevant molecules, such as DNA, RNA, proteins, carbohydrates and unsaturated fatty acids of the cell membranes [2-5]. Oxidative stress may not be considered an epiphenomenon in the critically ill patient but part of the underlying pathophysiologic events leading to mitochondrial dysfunction and the systemic inflammatory response syndrome (SIRS), which can lead to multiple organ dysfunction syndrome (MODS) [6].

The antioxidant endogenous defense system in humans consists of a variety of extracellular and intracellular antioxidants which are able to protect tissues from reactive oxygen species (ROS) and reactive nitrogen species (RNS) induced injury [3]. Trace elements, such as copper, manganese, zinc, iron and selenium are required for the activity of superoxide dismutase (SOD), catalase and glutathione peroxidase (GPx), respectively. In addition, the non-enzymatic defense mechanisms include endogenous molecules (that is, glutathione, albumin) and vitamins (such as E, C and β-carotene) [2,3]. Low levels of endogenous vitamins and trace elements in SIRS are due to escape to the interstitial compartment by capillary leakage, hemodilution, previous insufficient intake, and continuous renal replacement therapies (CRRT) [7]. In the critically ill, the most severe cases of SIRS are associated with the most severe antioxidant depletion [8-10].

In the last two decades, several clinical trials have evaluated the role of antioxidant micronutrients as monotherapy or in combined therapy (enteral or parenteral antioxidant cocktails) as part of an antioxidant strategy for critically ill SIRS patients. These have been reviewed in prior meta-analyses but since these publications [11,12], additional RCTs have been reported [13-18]. The aim of the current study was to provide an up-to-date systematic review and meta-analysis on all randomized clinical studies of vitamins and trace elements as pharmaconutrient therapy on relevant clinical outcomes in critically ill patients. In addition, we conducted several hypothesis-generating subgroup analyses to illuminate the optimal methods of administering antioxidants.

## Materials and methods

### Study identification

We conducted a systematic review of the published literature to identify all relevant clinical trials using text word or MeSH headings containing "randomized," "blind," "clinical trial," "nutritional support", "enteral nutrition", "parenteral nutrition", antioxidants," "vitamins", "trace elements", "selenium", "zinc", "copper", "manganese", "vitamins A, C and E", "critical illness" and "critically ill". To locate these articles we performed computerized searches on MEDLINE, EMBASE, CINAHL the Cochrane Controlled Trials Register, and the Cochrane Database of Systematic Reviews) from 1980 to December 2011. We also searched our personal files and comprehensive review articles were searched for additional original studies. No language restrictions were placed on the searches. Abstracts from scientific meetings were accepted for inclusion into this systematic review if a copy of the manuscript was available to complete the abstraction form.

### Study selection criteria

We only included original studies if they met the following inclusion criteria: a) study design: randomized clinical trials (RCTs); b) population: critically ill adult patients (>18 years of age); c) intervention: trace elements and/or vitamins versus placebo (either via enteral, parenteral, or both); d) study outcomes: must have included one of the following: mortality, intensive care unit (ICU) and hospital length of stay (LOS), infectious complications, and other clinically important complications. We excluded the clinical studies that reported only biochemical, metabolic, immunologic or nutritional outcomes. We have excluded trials that supplemented N-acetylcysteine (NAC) [19,20] in addition to trace elements and vitamins because this amino acid has shown to be potentially harmful in critically ill SIRS patients, particularly when it is started 24 hours after hospital admission [21].

Additionally, we excluded clinical studies that supplemented selected substrates, such as glutamine, arginine and omega-3 fatty acids, as pharmaconutrients in immune-enhancing diets (IEDs) in addition to vitamins and trace elements. Critically ill patients were defined as patients admitted to an ICU. When this was unclear, we considered a mortality rate higher than 5% in the control group to be consistent with critical illness.

All original studies were abstracted in duplicate independently by two reviewers, using a data abstraction form with a scoring system (Additional file 1 Table s1), which has been used previously [22]. Disagreement in the individual scores of each of the categories was resolved by consensus between both reviewers. We attempted to contact the authors of included studies and requested additional information not contained in published articles. We scored the methodological quality of individual trials considering the following key features of high-quality studies: a) extent to which randomization was concealed, b) blinding, c) analysis was based on the intention-to-treat (ITT) principle, d) comparability of groups at baseline, e) extent of follow-up, f) description of treatment protocol and co-interventions, and g) definition of clinical outcomes. Each individual study was scored from 0 to 14 (Table 1).

### Data synthesis

The primary outcome of the systematic review was overall mortality. From all studies, we combined hospital mortality where reported (specified or assumed to be hospital if not specified). If hospital mortality was not reported, we used ICU mortality or, if ICU mortality was not reported, we used 28-day mortality. Secondary outcomes included infection and ICU and hospital LOS. We used definitions of infections as defined by the authors in their original papers. If studies had more than one experimental intervention, these were each considered separately. We combined data from all trials to estimate the pooled risk ratio (RR) with 95% confidence intervals for death and infectious complications and overall weighted mean difference (WMD) with 95% confidence intervals for LOS data. All analyses, except the test for asymmetry, were conducted using Review Manager (RevMan) 5.1 software. (The Nordic Cochrane Centre, The Cochrane Collaboration, Copenhagen, Denmark, 2011) [23]. Pooled RRs were calculated using the Mantel-Haenszel estimator and WMDs were estimated by the inverse variance approach. The random effects model of DerSimonian and Laird was used to estimate variances for the Mantel-Haenszel and inverse variance estimators [24]. RRs are undefined and excluded for studies with no event in either arm. When possible, studies were aggregated on an intention-to-treat basis (Table 2). The presence of heterogeneity was tested by a weighted Mantel-Haenszel chi-square test and quantified by the I2 statistic as implemented in RevMan 5.1 [25]. The possibility of publication bias was assessed by generating funnel plots and testing asymmetry of outcomes using methods proposed by Rucker and colleagues [26]. We considered *P *<0.05 to be statistically significant and *P *<0.20 as the indicator of trend.

### A priori hypotheses testing

Given the larger number of trials and the heterogeneity of trial design, we performed several pre-specified, hypothesis-generating subgroup analysis to attempt to elucidate potentially more beneficial treatment strategies. Initially, we compared the results of trials that provided antioxidants via the enteral route compared to trials that provided intravenous antioxidants. Studies that have supplemented antioxidants using both routes (enteral and parenteral) were excluded from the subgroup analyses of parenteral versus enteral administration. Given that some trials showed that antioxidants, and particularly selenium, may offer some advantage in the most seriously ill patients, we compared studies of patients with higher mortality in the control group vs. lower mortality. When we considered the distribution of control group mortality rates, there was a clustering of studies with a mortality <10% and then a clustering with a control groups mortality >23%. These clusters were used in our subgroup analysis. According to the previous meta-analysis [11], selenium was considered the cornerstone of antioxidant therapy and we compared trials that utilized selenium in their antioxidant strategy versus those that did not. Of those trials that utilized selenium, we compared those that administered the selenium via the parenteral route compared to the enteral route. Of those trials that utilized intravenous selenium, we considered the following additional exploratory subgroup analyses: a) trials that use selenium (monotherapy) by itself versus combined with other nutrient (combination therapy); b) trials that utilized an intravenous rapid bolus loading dose of selenium versus those that did not; c) trials that provided parenteral selenium via continuous infusion versus parenteral selenium via intermittent bolus; and finally d) studies that utilized a selenium daily dose lower than 500 μg, equal to 500 μg, and greater than 500 μg.

## Results

### Study identification and selection

A total of 55 relevant citations were identified from the search of computerized bibliographic databases and a review of reference lists from related articles. Of these, we excluded 34 due to the following reasons: 14 trials did not include ICU patients [27-40]; 4 trials did not evaluate clinically important outcomes [41-44]; 7 trials studied nutrients other than micronutrients (vitamins and trace elements) [19,20,45-49]; 3 trials were duplicated publications of included trials [50-52]; 2 were meta-analysis or systematic reviews [15,53]; and 3 additional trials were excluded because one was published only as an abstract without possibility to access the full article [54] and one was pseudorandomized [55]. In the end, 21 RCTs including 2,531 patients met the inclusion criteria and were included in this systematic review [13-18,56-70] (Additional file 2, Table s2; Additional file 3, Table s3). The authors reached 100% agreement for inclusion of relevant trials in this review. The mean methodological score of all trials was 8.3 (range 4 to 13) of a maximum of 14. Randomization was concealed in 5/20 (24%) trials, ITT analysis was performed in 12/20 (60%) trials and 8/20 (40%) trials were double blinded.

### Meta-analyses of primary outcome

#### Overall effect on mortality

**Figure 1:**
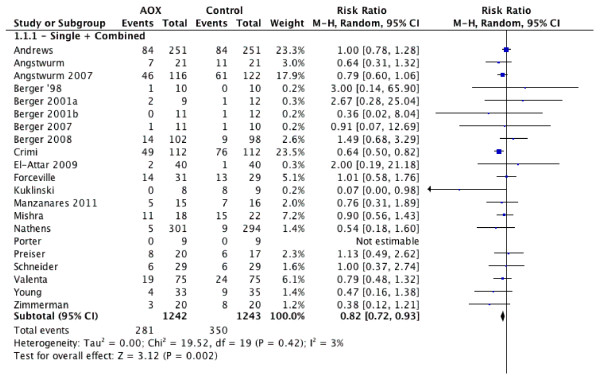
**Effects of antioxidant strategies on mortality (n = 20)**. AOX, antioxidants; RR, risk ratio; 95% CI, 95% confidence intervals.

When the results of 20 RCTs [13-18,56-70] that evaluated mortality as one of the outcomes were statistically aggregated, overall antioxidant micronutrients were associated with a significant reduction in mortality (risk ratio (RR) = 0.82, 95% confidence intervals (CI) 0.72 to 0.93, *P *= 0.002; see Figure 1). The test for heterogeneity was not significant (*P *= 0.42, I^2 ^= 3.0%). A funnel plot (data not shown) was created and the test of asymmetry was not significant (*P *= 0.35).

### Secondary outcomes

#### Overall effect on infectious complications

**Figure 2:**
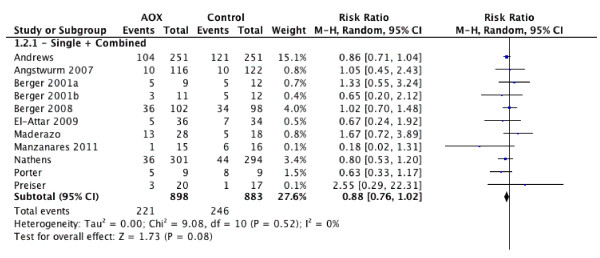
**Effect of antioxidants on infections (n = 10)**. AOX, antioxidants; RR, risk ratio; 95% CI, 95% confidence intervals.

Ten trials [13,14,16,17,57,62-65,67] reported overall infections and when these data were aggregated, antioxidant strategies were associated with a trend towards a reduction in infectious complications in critically ill patients (RR= 0.88, 95% CI 0.76 to 1.02, *P *= 0.08, see Figure 2). The test for heterogeneity was not significant (*P *= 0.52, I^2 ^= 0%).

#### Overall effect on length of stay

When the nine trials [13,16,18,60,62,64,67,68,70] that reported on ICU LOS were aggregated, antioxidants had no effect in LOS WMD = 0.07, 95% CI -0.08, 0.22, *P *= 0.38). Furthermore, there was no effect on hospital LOS when the data from five trials [13,16,60,62,64] reporting on this outcome were aggregated (WMD = -0.13, 95% CI -0.35 to 0.09, *P *= 0.25; test for heterogeneity was not significant *P *= 0.73, I^2 ^= 0%).

#### Overall effect on ventilator days

**Figure 3:**
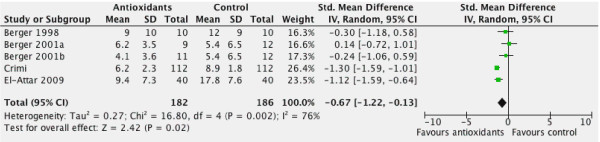
**Effect of combined antioxidant therapy on ventilation days (n = 4)**. RR, risk ratio; 95% CI, 95% confidence intervals.

When the four trials [14,60,64,66] that reported ventilator days were aggregated, antioxidants significantly decreased the duration of mechanical ventilation (WMD = -0.67, 95% CI -1.22 to -0.13, *P *= 0.02, see Figure 3).

### Subgroup analysis

#### Parenteral/Intravenous versus enteral route

**Figure 4:**
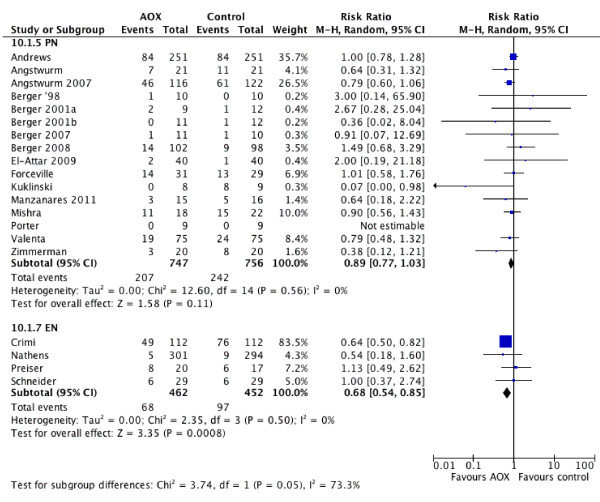
**Effect of combined antioxidant by parenteral (n = 15) and enteral route on mortality (n = 4)**. AOX, antioxidants; EN: enteral nutrition; PN: parenteral nutrition; RR, risk ratio; 95% CI, 95% confidence intervals.

Antioxidants were supplemented by parenteral route in 15 [13-17,57,59-62,64,67-70] of the 21 trials; 1 trial [65] was excluded because it supplemented vitamin E by enteral route and vitamin C by parenteral route. When the results of the trials using antioxidants via parenteral route were aggregated, antioxidants were associated with a trend towards reduction in mortality (RR = 0.89, 95% CI 0.77 to 1.03, *P *= 0.11); test for heterogeneity was not significant *P *= 0.56, I^2 ^= 0%). Meanwhile, four trials [18,63,65,66] that evaluated antioxidants by enteral route were associated with a significant reduction in mortality (RR = 0.68, 95% CI 0.54 to 0.85, *P *= 0.0008; test for heterogeneity *P *= 0.50, I^2 ^= 0%, see Figure 4). The test for subgroup differences was borderline significant, *P *= 0.05, I^2 ^= 0%. Seven trials [13,14,17,57,62,64,67] using intravenous antioxidants evaluated infectious complications. When aggregated these studies showed that antioxidant supplementation by intravenous route was associated with a trend towards reduced infections (RR = 0.89, 95% CI 0.76 to 1.03, *P *= 0.12; test for heterogeneity was not significant *P *= 0.57, I^2 ^= 0%). Only one RCT [63] reported the effects of enteral antioxidants on infections, so meta-analysis was not done.

#### Higher vs. lower mortality

**Figure 5:**
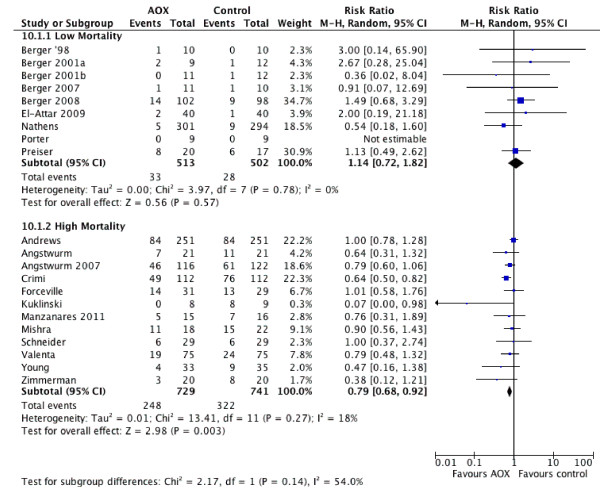
**Effects of antioxidants supplementation on mortality according to high or low mortality in the control group**. AOX, antioxidants; RR, risk ratio; 95% CI, 95% confidence intervals.

Subgroup analyses showed that antioxidant supplementation was associated with a significant reduction in overall mortality among patients with higher risk of death [15-18,56,58,59,61,66,67,69,70] (>10% mortality in the control group) (RR 0.79, 95% CI 0.68 to 0.92, *P *= 0.003). There was no significant effect observed for the trials of patients with a lower mortality in the control group [13,14,60,62-65,68] (RR = 1.14, 95% CI 0.72 to 1.82, *P *= 0.57). The test for subgroup differences was not significant (*P *= 0.14, I^2 ^= 54%, see Figure 5). Six trials with low mortality in the control group [13,14,62-65] showed no effect on infections (RR = 0.87, 95% CI 0.69 to 1.10, *P *= 0.25). Furthermore, four trials with higher mortality in the control group [16,17,57,67] did not show effect on infections (RR = 0.95, 95% CI 0.60 to 1.49, *P *= 0.81). The test for subgroup differences was not significant (*P *= 0.76).

#### Selenium versus non-selenium

There were 16 trials [13-18,56,59-62,65,67-70] that evaluated selenium alone or combined with other micronutrients in antioxidant cocktails. When aggregated, selenium supplementation was associated with a trend toward a reduction in mortality (RR = 0.89, 95% CI 0.77 to 1.03, *P *= 0.12). When trials that did not use selenium were aggregated [58,63,65,66], there was a significant reduction in mortality (RR = 0.66, 95% CI 0.52 to 0.82, *P *= 0.0003). The test for subgroup differences was significant (*P *= 0.03). The seven trials using selenium [13,14,16,17,62,64,67] demonstrated a trend toward a reduction in infections (RR = 0.87, 95% CI 0.74 to 1.02, *P *= 0.08) whereas three trials not using selenium [57,63,65] had no effect on infectious complications (RR = 1.10 95% CI 0.60 to 2.04, *P *= 0.75). The test for subgroup differences was not significant (*P *= 0.46).

#### Parenteral vs. enteral selenium

Next, we compared those trials that administered the selenium via the parenteral route versus the enteral route. There were 15 trials that evaluated parenteral selenium [13-18,56,59-61,64,67-70]. When aggregated, parenteral selenium supplementation was associated with a trend toward a reduction in mortality (RR = 0.89, 95% CI 0.77 to 1.03, *P *= 0.11). Furthermore, seven trials [13,14,16,17,62,64,67] using parenteral selenium demonstrated a trend toward a reduction in infections (RR = 0.87, 95% CI 0.74 to 1.02, *P *= 0.08). We could not meta-analyze the enteral subgroup because only one RCT [18] using enteral selenium reported on mortality, infections, ICU and hospital LOS.

#### Parenteral selenium subgroup analyses

##### Selenium monotherapy versus selenium combined

**Figure 6:**
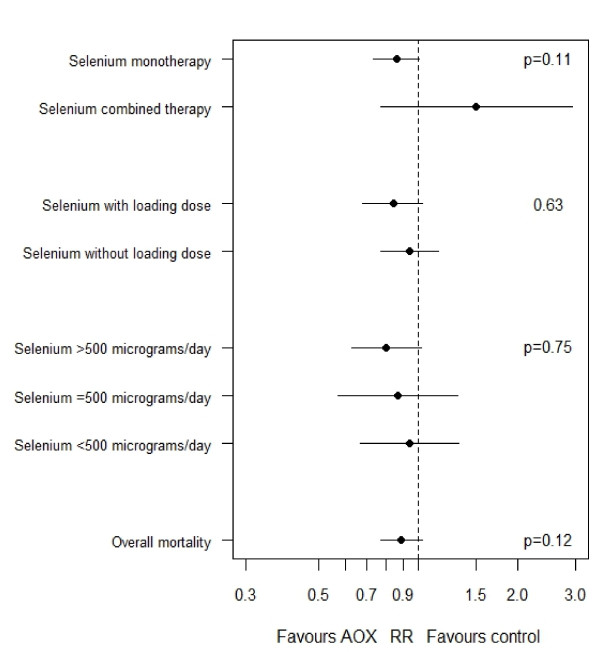
**Results of subgroup analyses examining the effect of parenteral selenium supplementation on mortality**. RR, risk ratio. *P*-values refer to the differences in the effects of selenium on mortality between subgroups.

Nine RCT [15-17,56,59,61,67,69,70] have supplemented selenium as monotherapy. When we aggregated these studies, selenium supplementation showed a trend toward reduction in mortality (RR = 0.86, 95% CI 0.73 to 1.01, *P *= 0.06). When the effect on mortality of five trials [13,60,62,64,68] using selenium in combined therapy was evaluated, parenteral antioxidants cocktails with selenium had no effect on mortality (RR = 1.50, 95% CI 0.77 to 2.94, *P *= 0.23). The test for subgroup differences tended towards statistical significance (*P *= 0.11, see Figure 6).

**Figure 7:**
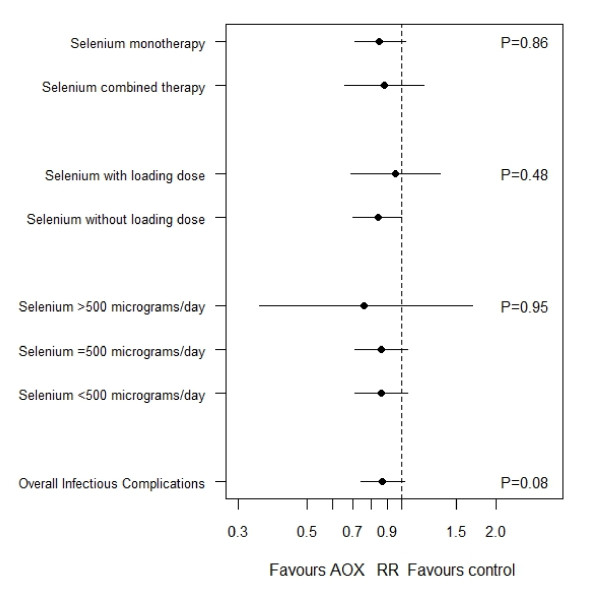
**Results of subgroup analyses examinating the effects of parenteral selenium supplementation on infections**. RR, risk ratio. *P*-values refer to the differences in the effects of selenium on infectious complications between subgroups.

The effect of selenium monotherapy on infections was evaluated in three trials [16,17,67] showing a trend towards reductions in infectious complications (RR = 0.85, 95% CI 0.71 to 1.03, *P *= 0.10). Meanwhile, four RCTs [13,14,62,64] evaluated combined therapy and showed no effect on infections (RR = 0.88, 95% CI 0.66 to 1.18, *P *= 0.40); test for subgroup differences was not significant (*P *= 0.86, see Figure 7).

##### Intravenous loading dose versus no loading dose

Parenteral selenium via loading dose as a bolus was evaluated in five RCTs [13,15,16,59,67]. When these studies were aggregated, selenium supplementation with a bolus showed a trend toward reduction in a mortality (RR = 0.81, 95% CI 0.65 to 1.02, *P *= 0.07; test for heterogeneity *P *= 0.40, I^2 ^= 1%). On the other hand, parenteral selenium without loading dose was evaluated in 10 trials [14,17,56,60-62,64,68-70] and did not show effect on mortality (RR = 0.95, 95% CI 0.78 to 1.15, *P *= 0.59). The test of subgroup effects was not significant (*P *= 0.63, see Figure 6).

The effect of selenium loading dose on infections was evaluated in three trials [13,16,67] and showed no effect in infectious complications (RR = 0.96, 95% CI 0.69 to 1.33, *P *= 0.80). Meanwhile, four trials [14,17,62,64] evaluated parenteral selenium without loading dose and showed a borderline effect on infections (RR 0.84, 95% CI 0.70 to 1.00, *P *= 0.05); test for subgroup differences was not significant (*P *= 0.48, see Figure 7).

##### Selenium high dose versus lower dose

Four trials [16,59,67,69] using higher doses than a daily dose of 500 μg showed a trend towards a lower mortality (RR = 0.80, 95% CI 0.63 to 1.02, *P *= 0.07). The four trials [15,17,56,64] using doses equal to 500 μg had a smaller treatment effect that was not statistically significant (RR = 0.87, 95% CI 0.57 to 1.32, *P *= 0.51). Meanwhile, the seven trials [13,14,60-62,68,69] using doses lower than 500 μg had no effect on mortality (RR = 0.94, 95% CI 0.67 to 1.33, *P *= 0.75). Although numerically different, these effects' size differences are clinically important, but the test for subgroup differences was not significant (*P *= 0.75, see Figure 6).

Two trials [16,67] using doses higher than of 500 μg/d showed no effect on infections (RR = 0.76, 95% CI 0.35 to 1.69, *P *= 0.51). The two trials [17,64] using doses equal to 500 μg/d showed a trend towards a lower infections (RR = 0.86, 95% CI 0.71 to 1.05, *P *= 0.13) and the three trials [13,14,62] that used doses lower than 500 μg/d had no effect on infections (RR = 0.87, 95% CI 0.64 to 1.19, *P *= 0.39). The test for subgroup differences was not significant (*P *= 0.95, see Figure 7).

## Discussion

Critical illness is associated with a significant redox imbalance which leads to mitochondrial dysfunction, SIRS and MODS [1,6]. In this context, it may be that supplemental trace elements and vitamins represent an important therapeutic option for critically ill patients. We have systematically reviewed 21 eligible RCTs in ICU patients for evaluating the effects of combined antioxidants (vitamins and trace elements) where the nutrients are provided dissociated from standard nutrition. With the exception of six larger trials [13,15,17,65-67], most RCTs included in this systematic review were relatively small studies with the number of patients less than 100, and thus inadequate to detect clinically important treatment effects of combined antioxidants on mortality. The advantage of meta-analytic techniques is that they can combine across studies to gain a more precise treatment effect. When they were statistically aggregated, we found a significant reduction in mortality and mechanical ventilation days and a trend towards reduced infections with no overall effect on ICU or hospital LOS in critically ill patients. Since the mortality effect is greater than the effect on infectious complications, it is plausible that the mortality effect could be mediated by different mechanisms related to improvement of organ failure, not by reducing infection, although this is only a postulate and not supported by our data. Furthermore, given the wide variety of clinical diagnoses and the heterogenous population of ICU patients included in this systematic review (sepsis, severe sepsis/septic shock, trauma, burns, pancreatitis, head injury and SIRS) the results and conclusions may be applied to a broad, heterogeneous group of critically ill patients.

The presence of clinical and statistical heterogeneity in this meta-analysis enables us to explore the sources of heterogeneity and illuminate strategies for optimizing the treatment effect of antioxidants in critically ill patients. Accordingly, we conducted several hypothesis-generating analyses. We observed that enteral antioxidants had a larger treatment effect on mortality (RR = 0.68 vs. 0.89). Notwithstanding, the data supporting enteral antioxidants are sparse and we have not really evaluated all end points comprehensively. Furthermore, the mortality effect is largely driven by the Crimi *et al*. [66] trial, a large RCT (n = 216) that showed that antioxidant supplementation with vitamins C (500 mg/d) and E (400 UI/d) in enteral feeding for 10 days is associated with decreased 28-day mortality (45.7% versus 67.5% *P *<0.05) [66]. This trial explains 83.5% of the signal and is thus a very unstable estimate. In addition, high mortality observed in the regular feeding group questions the generalizabily of this study. Finally, we still show a possible treatment effect with parenteral antioxidants on infectious complications. Therefore, we do not conclude that enteral antioxidants are better but would suggest that either route of administration is acceptable given our current knowledge. In fact, there is a rationale for combining enteral with parenteral antioxidants to maximize the likelihood of treatment effect [71].

We have demonstrated a more pronounced effect in reducing mortality with antioxidant strategies in the most seriously ill ICU patients. In fact, when we aggregated the data across the RCTs using a mortality cut off value of 10% in the control group, we found a significant effect of antioxidant supplementation in RCT with a mortality higher than 10% (*P *= 0.003). This finding supports the notion that patients with more severe insults and higher mitochondrial dysfunction resulting from bioenergetic failure exhibited the largest depletion of antioxidants [72]. Therefore, these patients may exhibit greater improvement with antioxidant supplementation compared to less sick patients.

In another subgroup analysis, antioxidant strategies without selenium were associated with a significant reduction in mortality but no effect on infectious complications. Nevertheless, data are sparse and once again the strength of that estimation is derived from the Crimi *et al*. study [66], which explains the 84% of the signal; hence, this estimate is very unstable. On the other hand, there are 16 RCTs that evaluated selenium-based strategies and when these results are statistically aggregated, we observed a trend towards reduced mortality (RR = 0.89, 95% CI 0.77 to 1.03, *P *= 0.12) and infection (RR = 0.87, 95% CI 0.74 to 1.02, *P *= 0.08). Nothwistanding, current knowledge shows that selenium exhibits antioxidant, antiinflammatory and immunomodulatory effects [73]. In this context, selenium has been shown to inhibit the activation of nuclear factor kappa-B (NF-kB) by controlling selenoprotein genes expression [74,75]. Likewise, selenium suppresses C reactive protein synthesis and increases release of L-selectin from monocytes while decreasing soluble L-selectin, which has been reported to be associated with high mortality in septic patients [76]. These mechanisms are likely to contribute to the modulatory effects of selenium on the inflammatory response [75]. Therefore, we certainly believe that selenium cannot be left out of antioxidant treatment strategies in the critically ill.

We observed considerable variation in how selenium is administered in the included RCTs and, thus, we conducted several additional subgroup analyses to evaluate whether we can optimize the treatment effect of selenium-based strategies. We first compared studies that evaluated selenium as monotherapy and compared them to studies of combination antioxidant therapy that included selenium. We observed important trends towards reduced mortality (RR = 0.86, 95% CI 0.73 to 1.01, *P *= 0.06) and reduced infectious complications (RR = 0.85, 95% CI 0.71 to 1.03, *P *= 0.10) associated with selenium monotherapy, and no evidence of a treatment effect was associated with combination therapy. These data support the notion that selenium could be the cornerstone of antioxidant strategies [11]; however, in this meta-analysis we have previously demonstrated that non-selenium-based studies are also associated with a significant positive treatment effect. Thus, we conclude that a combination of selenium with other trace elements and vitamins is probably warranted.

Current knowledge from animal studies shows that selenium given as an intravenous loading dose has a biphasic action; initially as a pro-oxidant and then as an antioxidant [77]. Nevertheless, the early transient pro-oxidant effect of selenite might be a useful therapeutic strategy for some ICU patients [78]. In early stages of SIRS, a selenium loading dose given as an intravenous bolus may be able to induce a direct reversible inhibition of NF-κB binding to DNA, controlling gene expression and thus down-regulating the synthesis of pro-inflammatory cytokines [76,79]]. Furthermore, in a sheep model of severe sepsis, the bolus of sodium selenite was able to improve hemodynamics, delaying arterial hypotension, improving cardiac index, with delayed hyperlactatemia, and fewer sepsis-induced microvascular alterations [80]. Unfortunately, these findings have never been proven in clinical trials. We explored the effect of selenium loading dose as an intravenous bolus given in a short time between 30 minutes and 2 hours. According to our results, a parenteral loading dose showed a trend towards reduction in mortality (RR = 0.84, 95% CI 0.68 to 1.03, *P *= 0.09) whereas studies that did not use a bolus loading dose did not show effect on mortality (RR= 0.94, 95% CI 0.77 to 1.15, *P *= 0.56). The absence of a significant test of subgroup differences weakens any inferences from this subgroup analysis but it remains a plausible hypothesis that studies that employ a bolus loading dose may have a greater mortality effect than those that did not.

In addition, we did not find any significant numerical difference among different selenium daily doses. However, trials using daily doses greater than 500 μg showed a trend towards a tendency to reduce mortality (*P *= 0.07). This finding is mostly due to the two German RCTs [56,67], in which using a daily dose of 1,000 μg showed a trend reduction in mortality.

The strength of our meta-analysis includes the fact that we have used several methods to reduce bias (comprehensive literature search, duplicate data abstraction, specific criteria for searching and analysis) and have focused on clinically important primary outcomes. Notwithstanding, we are aware that our meta-analysis has several limitations. Perhaps, the major limitation was the small number of trials included in certain subgroup analyses and the effect of one enteral, non-selenium RCT [66] on the mortality in the parenteral vs. enteral subgroups analysis and the selenium vs. non selenium subgroups analysis. Moreover, in those RCTs that have provided micronutrients as part of PN, such as SIGNET [17], which supplemented PN selenium, a difference between the prescribed and the provided dose is possible due to PN intolerance, which adds uncertainty about the true selenium daily dose.

In spite of these limitations, we have demonstrated that antioxidant supplementation in the critically ill may significantly reduce overall mortality and shorten ventilation days with a trend towards reduction in infectious complications. Nonetheless, many questions on antioxidant strategies in the ICU still remain unanswered. Further research is warranted to define the optimal combination, optimal dose and the timing of supplementation of micronutrients [9]. Although the optimal time to start antioxidants could not be determined from this meta-analysis, both experimental and clinical data support the concept that antioxidants are more effective when initiated prior to injury [3].

## Conclusions

In this meta-analysis, we have demonstrated that trace elements and vitamins as antioxidants may be able to significantly decrease mortality and shorten mechanical ventilation days and are associated with a trend towards reduced infectious complications in critically ill patients. The treatment effect may be greatest in patients with greater severity of illness. Furthermore, the therapeutic effect may also depend on the type of intervention and/or the method of administration. Antioxidant cocktails with intravenous selenium at high doses may optimize the therapeutic effect of antioxidant strategies. Further research to optimize the therapeutic effect of antioxidants is warranted.

## Key messages

• Critical illness is characterized by oxidative stress with antioxidant depletion. In this context, supplementation of antioxidant micronutrients could restore antioxidant status, improving clinical outcomes.

• Trace elements and vitamins, as antioxidants, may significantly decrease mortality and shorten mechanical ventilation days in ICU patients.

• Antioxidant micronutrients strategies are associated with a trend towards a reduction in infections.

• The treatment effect may be greatest in patients with greater severity of illness.

• Antioxidant cocktails with intravenous selenium at a daily dose higher than 500 μg may optimize the therapeutic effect of antioxidant strategies.

## Abbreviations

AOX: antioxidants; CI: confidence interval; COPD: chronic obstructive pulmonary disease; C.Random: concealed randomization; CRRT: continuous renal replacement therapies; D5W: dextrose 5% in water; EN: enteral nutrition; GPx: glutathione peroxidase; HAP: hospital acquired pneumonia; ICU: intensive care unit; IEDs: immune-enhancing diets; ITT: intention to treat; IV: intravenous; LOS: length of stay; MODS: multiple organ dysfunction syndrome; N: number of patients; NA: non-attributable; NAC: N-acetyl cysteine; NF-kappaB: nuclear factor kappa-B; PN: parenteral nutrition; RCT: randomized controlled trials; RNS: reactive nitrogen species; ROS: reactive oxygen species; RR: risk ratio; SIRS: systemic inflammatory response syndrome; SOD: superoxide dismutase; TBSA: total body surface area; VAP: ventilator associated pneumonia; WMD: weighted mean difference

## Competing interests

The authors declare that they have no competing interests.

## Authors' contributions

All authors read and approved the final manuscript. WM and DKH are responsible for writing the manuscript. XL and LM are responsible for the statistics. RD, WM and DKH are responsible for the data collection.

## Supplementary Material

Additional file 1 **Table 1. Methodological scoring system**. Click here for file

Additional file 2 **Table 2. Details of included trials**. Study designs of randomized trials evaluating antioxidant micronutrients in critically ill patients. COPD, chronic obstructive pulmonary disease; C.Random, concealed randomization; D5W, dextrose 5% in water; EN, enteral nutrition; ICU, intensive care unit; ITT, intention to treat; IV, intravenous; N, number of patients; PN, parenteral nutrition; SIRS, systemic inflammatory response syndrome; TBSA, total body surface area. Click here for file

Additional file 3 **Table 3. Outcomes of included trials**. Results of randomized clinical trials evaluating antioxidant micronutrients in critically ill patients. COPD, chronic obstructive pulmonary disease; C.Random, concealed randomization; EN, enteral nutrition; HAP, hospital acquired pneumonia; Hosp, hospital; ICU, intensive care unit; ITT, intent to treat; IV, intravenous; NA, non-attribuible; NR, non-reported; PN, parenteral nutrition; SIRS, systemic inflammatory response syndrome; TBSA, total body surface area; VAP, ventilator associated pneumonia. Click here for file
