# Supplementary material for: Antioxidant micronutrients in the critically ill: a systematic review and meta-analysis
Source: Crit Care. 2012 Apr 25;16(2):R66. doi: 10.1186/cc11316 (PMC3681395; doi:10.1186/cc11316)
Supplement: Additional file 1 — Table 1. Methodological scoring system. [file cc11316-S1.DOC]

|  | **Score** | | |
| --- | --- | --- | --- |
|  | **0** | **1** | **2** |
| Randomization | … | Not concealed or not sure | Concealed randomization |
| **Analysis** | Other | … | Intention to treat |
| **Blinding** | Not blinded | Single blind | Double blinded |
| **Patient selection** | Selected patients or unable to tell | Consecutive eligible patients | ….. |
| **Comparability of groups at baseline** | No or not sure | Yes | …. |
| **Extent of follow-up** | < 100% | 100% | …. |
| **Treatment protocol** | Poorly described | Reproducibly described | …. |
| **Co-interventions** | Not described | Described but not equal or not sure | Well described and all equal |
| **Outcomes** | Not described | Partially described | Objectively defined |
