# Supplementary material for: Antioxidant micronutrients in the critically ill: a systematic review and meta-analysis
Source: Crit Care. 2012 Apr 25;16(2):R66. doi: 10.1186/cc11316 (PMC3681395; doi:10.1186/cc11316)
Supplement: Additional file 2 — Table 2. Details of included trials. Study designs of randomized trials evaluating antioxidant micronutrients in critically ill patients. COPD, chronic obstructive pulmonary disease; C.Random, concealed randomization; D5W, dextrose 5% in water; EN, enteral nutrition; ICU, intensive care unit; ITT, intention to treat; IV, intravenous; N, number of patients; PN, parenteral nutrition; SIRS, systemic inflammatory response syndrome; TBSA, total body surface area. [file cc11316-S2.DOC]

**Table 2 Details of Included Trials**

| **Study** | **Population** | **Methods score** | **Intervention** |
| --- | --- | --- | --- |
| **Kuklinski et al 1991** | Patients with acute pancreatic necrosis N = 17 | C. Random: not sure  ITT: no Blinding: no (4) | PN + selenium supplementation (500 μg /d) vs. PN without selenium supplementation |
| **Maderazo et al, 1991** | Blunt Trauma N = 46 | C. Random: yes  ITT: yes Blinding: double (7) | 200 mg Ascorbic acid, then increase to 500 mg + 50 mg α tocopherol in 100 ml of D5W vs. 100 ml of D5W (Experimental group divided into 2 groups, 200 mg ascorbic acid vs. 50 mg α tocopherol) .Given as 2 hr infusions from Day 0-7. (All groups received enteral nutrition or po intake) |
| **Young et al, 1996** | Severely head injured patients, ventilated N = 68 | C. Random: yes  ITT: yes Blinding: double (7) | 12 mg elemental zinc via PN, then progressing to oral zinc from 0- 15 days vs. 2.5 mg elemental zinc, then progressing to oral placebo |
| **Zimmerman et al, 1997** | Patients with SIRS, APACHE > 15 and multiorgan failure score >6  N = 40 | C. Random: no  ITT: yes Blinding: no (6) | IV Selenium as sodium selenite 1000 μg as a bolus and then 1000μg sodium selenite 24 hrs as a continuous infusion over 28 days vs. standard |
| **Berger et al, 1998** | Burns > 30 % TBSA N = 20 | C. Random: yes  ITT: yes Blinding: double blind (12) | IV Copper (40.4 μmol), selenium (159 μg), zinc (406 μmol) + standard trace elements vs. standard trace elements (Copper 20 μmol, selenium 32 μg, zinc 100 μmol) from day 0- 8, all received early EN |
| **Porter et al, 1999** | Surgical ICU Penetrating trauma patients with injury severity score ≥ 25 N = 18 | C. Random: yes  ITT: yes Blinding: no (9) | 50 μg selenium IV q 6 hrs + 400 IU Vit E, 100 mg Vit. C q 8 hrs and 8 g of N-acetylcysteine (NAC) q 6 hrs via nasogastric or oral route, from Day 0-7 vs. none |
| **Angstwurm et al, 1999** | Patients with systematic inflammatory response syndrome from 11 ICUs N = 42 | C. Random: not sure  ITT: yes Blinding: no (10) | PN with high dose selenium (535 μg x 3 days, 285 μg x 3 days and 155 μg x 3 days and 35 μg thereafter) vs. low dose selenium (35 μg/day for duration of study) |
| **Preiser et al, 2000** | Mixed ICU N = 51 | C. Random: not sure  ITT: no Blinding: single (7) | Antioxidant rich formula via EN (133 μg /100 ml vit. A, 13 mg/100 ml Vit C & 4.9 mg/100 ml Vit E) vs. isonitrogenous, isocaloric standard formula (67 μg /100 ml vit. A, 5 mg/100 ml Vit C and 0.81 mg/100 ml Vit E) from Day 0- 7 |
| **Berger et al, 2001** | Trauma patients, surgical ICU  N = 32 | C. Random: yes  ITT: no Blinding: double blind (9) | IV Selenium supplementation (500 μg/day ) vs. placebo (Selenium group randomized further to two groups: 500 μg Selenium alone vs. 500 μg Selenium + 150 mg α tocopherol + 13 mg zinc) given slowly for 1^st^ 5 days after injury (All groups received EN) |
| **Nathens et al, 2002** | General Surgical/Trauma ICU  N=770 | C.Random: not sure  ITT: no Blinding: no (7) | α tocopherol 1000 IU q 8 h via naso or orogastric tube and ascorbic acid 1000 mg q 8 h via IV vs. standard care |
| **Berger et al, 2007** | Burns > 20 % TBSA  N = 21 | C.Random: not sure  ITT: yes Blinding: no (8) | IV 100 ml of Copper (59 μmol) + Selenium (375 μgm + zinc (574 μmol) vs. NaCl (0.9%) from admission for 5-15 days. Both groups were on EN. |
| **Crimi et al, 2004** | Mixed ICU  N = 224 | C.Random: not sure  ITT: no Blinding: no (7) | Vit C (500 mg), Vit E (400 IU) within 72 hrs for 10 days vs. isotonic saline (all groups received EN) |
| **Angstwurm 2007** | Multicentre mixed ICUs  N =249 | C.Random: not sure  ITT: no Blinding: double (8) | 1000μg Selenium IV within 1 hr followed by 1000μg Selenium for 14 days vs. NaCl (0.9%) (all patients received EN or PN) |
| **Forceville 2007** | Septic shock patients from 7 ICUs N = 60 | C.Random: not sure  ITT: no Blinding: double (8) | 4000μg Selenium IV on day 1 followed by 1000μg Selenium for 9 days vs. NaCl (0.9%) (all patients received EN or PN) |
| **Mishra et al, 2007** | Septic ICU patients  N = 40 | C.Random: not sure  ITT: yes Blinding: double (9) | 474 μg Selenium IV x 3 days followed by 316 μg x 3 days, 158 μg x 3 days and 31.6 μg thereafter vs. 31.6 μg Selenium (all patients received EN or PN). |

| **Berger et al, 2008** | Mixed ICU  N = 200 | C.Random: not sure  ITT: yes Blinding: no (10) | IV Selenium supplementation loading dose 540 μg/day + zinc (60 mg) + Vit C 2700 mg + Vit B 305 mg + Vit E enteral 600 mg + Vit E 12.8 mg IV for 2 days followed by half the dose of all vs. standard vitamins. (All groups received EN or PN) |
| --- | --- | --- | --- |
| **El-Attar et al, 2009** | COPD patients  N= 80 | C.Random: yes  ITT: yes Blinding: yes (12) | IV selenium as sodium selenite 100 μg/day, zinc 2 mg/day and manganese 0.4 mg/day. TE were administered during the period on mechanical ventilation |
| **Valenta et al, 2011** | Patients with sepsis or SIRS  N=150 | C. Random: not sure  ITT: yes Blinding: no (8) | IV Selenium supplementation loading dose 1000 μg on day 1 followed by 500μg/day for 5-14 days + <75μg/day of Na-selenite added to PN. vs. NaCl + <75μg/day of Na-selenite added to PN. |
| **Manzanares et al, 2011** | Septic or trauma patients  N=31 | C. Random: not sure  ITT: no (except mortality)  Blinding: single blind (9) | IV Selenium supplementation loading dose 2000 μg (2 hours) on day 1 followed by 1600μg/day for 10 days vs. NaCl as placebo |
| **Andrews et al, 2011** | Mixed ICU  N=502 | C. Random: yes  ITT: yes Blinding: double blind (13) | 500μg selenium supplemented PN (12.5g nitrogen, 2000kcal) vs. standard PN (12.5g nitrogen, 2000kcal) initiated after ICU admission (actual median 2.6 days) for 7 days (actual duration, mean 4.1 days). |
| **Schneider et al, 2011** | ICU patients with sepsis or SIRS  N = 58 | C.Random: not sure  ITT: yes Blinding: single blind (8) | Fresenius Kabi Intestamin (300μg selenium, zinc 20mg, vitamin C 1500mg, Vitamin E 500mg) vs. Fresubin original plus 250mL water delivered via duodenal tube and initiated within first 48h of ICU admission. Both groups received Fresenius Kabi original fiber and supplemental PN if <60% adequacy |

D5W: dextrose 5% in water; COPD: chronic obstructive pulmonary disease; C.Random: concealed randomization; EN: enteral nutrition; ICU: intensive care unit; ITT: intention to treat; IV: intravenous; N: number of patients; PN: parenteral nutrition; SIRS: systemic inflammatory response syndrome; TBSA: total body surface area.
