# Supplementary material for: Antioxidant micronutrients in the critically ill: a systematic review and meta-analysis
Source: Crit Care. 2012 Apr 25;16(2):R66. doi: 10.1186/cc11316 (PMC3681395; doi:10.1186/cc11316)
Supplement: Additional file 3 — Table 3. Outcomes of included trials. Results of randomized clinical trials evaluating antioxidant micronutrients in critically ill patients. COPD, chronic obstructive pulmonary disease; C.Random, concealed randomization; EN, enteral nutrition; HAP, hospital acquired pneumonia; Hosp, hospital; ICU, intensive care unit; ITT, intent to treat; IV, intravenous; NA, non-attribuible; NR, non-reported; PN, parenteral nutrition; SIRS, systemic inflammatory response syndrome; TBSA, total body surface area; VAP, ventilator associated pneumonia. [file cc11316-S3.DOC]

| **Study** | **Population** | **Mortality (%)** Experimental Control | | | **Infections (%)** Experimental Control | | | | LOS days **Experimental Control** | | |
| --- | --- | --- | --- | --- | --- | --- | --- | --- | --- | --- | --- |
| **Kuklinski et al, 1991** | Patients with acute pancreatic necrosis  N = 17 | ICU 0/8 (0) | ICU 8/9 ( 89) | | NA | NA | | | NR | NR | |
| **Maderazo et al, 1991*** | Blunt Trauma  N = 46 | NA | NA | | 13/28 (46) | 5/18 (28) | | | NR | NR | |
| **Young et al, 1996** | Severely head injured patients, ventilated  N = 68 | 4/33 (12) | 9/35 (26) | | NA | NA | | | NR | NR | |
| **Zimmerman et al, 1997** | Patients with SIRS, APACHE > 15 and multi  organ failure score >6  N = 40 | 3/20 (15) | 8/20 (40) | | NA | NA | | | NR | NR | |
| **Berger et al, 1998** | Burns > 30 % TBSA  N = 20 | 1/10 (10) | 0/10 (0) | | 1.9 ± 0.9 (1-4) per patient | 3.1 ± 1.1 (2-5) per patient | | | 30 ± 12 (10) **ICU**  54 ± 27 (10) **hospital** | 39 ± 13 (10) **ICU**  66 ± 31 (10) **hospital** | |
| **Porter et al, 1999** | Surgical ICU Penetrating trauma patients with injury severity score ≥ 25  N = 18 | 0/9 | 0/9 | | 5/9 (56) | 8/9 (89) | | | **ICU** 22 ± 25.2 Hospital 31.3 ± 23.4 | ICU 35.8 ± 21.9Hospital 49 ± 30 | |
| **Angstwurm et al, 1999** | Patients with systematic inflammatory response syndrome from 11 ICUs  N = 42 | **hospital** 7/21 (33) | **hospital** 11/21 (52) | | NA | NA | | | NR | NR | |
| **Preiser et al, 2000** | Mixed ICU  N = 51 | **ICU** 3/20 (15)  **hospital**  8/20 (40) | **ICU** 3/17 (18)  **hospital**  6/17 (35) | | 3/20 (15) | 1/17 (6) | | | 5 (3-26) | 5 (3-18) | |
| **Berger et al, 2001** | Trauma patients, surgical ICU  N = 32 | a) Selenium alone 2/9 (22)  **b) Selenium+zinc+α tocopherol 0/11 (0)** | 1/11 (9) | | a) Selenium alone 5/9 (56)  **b)Selenium +zinc+α tocopherol 3/11 (27)** | 5/12 (42) | | | **a) ICU** 8.0 ± 4.0 (9)  **Hospital** 82 ± 78 (9)  **b) ICU** 5.8 ±4.4 (11)  **Hospital** 60 ± 48 (11) | ICU 8.6 ± 8.1 (12) **Hospital** 64 ± 39 (12) | |
| **Study** | **Population** | **Mortality (%)** Experimental Control | | | **Infections (%)** Experimental Control | | | | LOS days **Experimental Control** | | |
| **Nathens et al, 2002** | General Surgical/Trauma ICU  N=596 | **ICU** 3/301 (1)  **Hospital** 5/301(2)  **28 day** 4/301 (1) | | **ICU** 9/294 (3)  **Hospita**l 9/294(3)  **28 day** 7/294 (2) | 36/301 (12) | | | 44/294 (15) | **ICU** 5.3 (mean)  **Hosp** 14.6 (mean) | | **ICU** 6.4 (mean)  **Hosp** 15.1 (mean) |
| **Berger et al, 2007** | Burns > 20 % TBSA N = 21 | 1/11 (9) | | 1/10 (10) | 2.1 ± 1.0 per patient | | | 3.6 ± per patient | **ICU** 35 ± 27 (11) | | **ICU** 47 ± 37 (10) |
| **Crimi et al, 2004** | Mixed ICU  N = 224 | **28 day**  49/112 (44) | | **28 day**  76/112 (68) | NA | | | NA | **Hospital** 26.5 (mean) | | Hospital 27.5 (mean) |
| **Angstwurm et al, 2007** | Multicentre mixed ICUs  N =249 | **28 day**  46/116 (40) | | **28 day**  61/122 (50) | **New infections (HAP)**  10/116 (9) 10/122 (8) | | | | **ICU** 15.1 ± 10 (116) | | **ICU** 12.7± 9 (122) |
| **Forceville et al, 2007** | Septic shock patients from 7 ICUs  N = 60 | **28 day**  14/31 (45)  **6 Month**  18/31 (59)  **1 year** 66% | | **28 day**  13/29 (45)  **6 Month**  20/29 (68)  **1 year** 71% | **Superinfection******  1/31 (3) 2/29 (7) | | | | **ICU**  21 (7-40)  **Hosp** 25 (7-68) | | ICU 18 (10-31) **Hosp** 33 (11-51) |
| **Mishra et al, 2007** | Septic ICU patients  N = 40 | **ICU** 8/18 (44)  **Hospital**  11/18 (61)  **28 day**  8/18 (44) | | **ICU** 11/22 (61)  **Hospital**  15/22 (68)  **28 day**  11/22 (50) | **Infections per patient**  1.5 ± 1.9 1.8 ± 1.6 | | | | **ICU**  21.3 ± 16.2 (18) | | ICU 20.8 ± 21.8 (18) |
| **Berger et al, 2008** | Mixed ICU  N = 200 | **ICU**  8/102 (8)  **Hospital**  14/102 (14)  **3 month**  14/602 (14) | | **ICU**  5/98 (5)  **Hospital**  9/98 (11)  **3 month**  11/98 (11) | 36/102 (35) | | 34/98 (35) | | **ICU** 5.8 ± 5.4 (102)  **Hosp** 23 ± 20 (102) | | ICU 5.4 ± 5.7 (98) **Hosp** 26 ± 20 (98) |
| **El-Attar et al, 2009** | ICU COPD patients  N= 80 | **ICU**  2/40 (5.6) | | **ICU**  1/40 (2.9) | **VAP**  5/36 (13.9%) | | **VAP**  7/34 (20.6%) | | NR | | NR |
| **Valenta et al, 2011** | Patients with sepsis or SIRS N=150 | **28-day**  19/75 (25) | | **28-day**  24/75 (32) | NR | | NR | | NR | | NR |
| **Manzanares et al, 2011** | Septic or trauma patients  N=31 | **ICU**  3/15 (20)  **Hospital**  5/15 (33) | | **ICU**  5/16 (31)  **Hospital**  7/16 (44) | **VAP**  3/15 (20%) | | **VAP**  7/16 (44%) | | **ICU** 14 ± 11 (15) | | **ICU** 13 ± 6 (16) |
| **Andrews et al, 2011** | Mixed ICU  N=502 | **ICU**  84/251 (33)  **6-month**  107/251 (43) | | **ICU**  84/251 (33)  **6-month**  114/251 (45) | **Confirmed**  104/251 (41) | | **Confirmed**  121/251 (48) | | **ICU** 13.2 (IQR 7.8- 23.7)  **Hosp** 29.8 (IQR 14.7-52.4) | | **ICU** 15.1 (IQR 8.3-28.4)  **Hosp** 31.2 (IQR 15.1-57.8) |
| **Schneider et al, 2011** | ICU patients with sepsis or SIRS  N = 58 | 6/29 (21) | | 6/29 (21) | **Secondary infection from day 8**  13/26 (50) | | **Secondary infection from day 8**  9/24 (38) | | **ICU** 29.8 ± 26 (29)  **Hosp** 44.4 ± 36.6 (29) | | **ICU** 26.5 ± 19.6 (29)  **Hosp** 47.2 ± 48.1 (29) |
